# Seroprevalence of Human Brucellosis in Wadi Al Dawaser region of Saudi Arabia

**DOI:** 10.12669/pjms.35.1.55

**Published:** 2019

**Authors:** Mohamudha Parveen Rahamathulla

**Affiliations:** Dr. Mohamudha Parveen Rahamathulla, PhD. Department of Medical Lab Sciences, College of Applied Medical Sciences, Prince Sattam bin Abdulaziz University, Wadi Al Dawaser-11991, Kingdom of Saudi Arabia

**Keywords:** Human brucellosis, Seroprevalence, ELISA, Saudi Arabia, Risk factors

## Abstract

**Background and Objective:**

Brucellosis is an important zoonotic disease and a major public health problem in the Middle East countries including Saudi Arabia. This study has evaluated the seroprevalence of human brucellosis in Wadi Al Dawaser region of Central Saudi Arabia.

**Methods:**

The study was conducted for three years (2015-2018) at Wadi Al Dawaser general hospital. A total of 6721 clinically suspected serum samples were collected over three years and tested by Rose Bengal Plate Test (RBPT), Serum Agglutination Test (SAT), IgM and IgG ELISA. A standard questionnaire to determine the risk factors were used among patients.

**Results:**

Of the total 6721 samples tested, 576 (8.6%) were seropositive for brucellosis. RBPT identified 427 (74.1%), SAT titers 493 (85.6%) cases. IgM ELISA and IgG ELISA were positive for 501 (86.9%) and 558 (96.8%) cases respectively. Among the positive cases, 77.3% were male with the male to female ratio of 3.3:1. Nearly, 82% were Saudi Nationals. Direct contact with domestic animals and consumption of raw milk were the risk factors identified. No seasonal variation was seen. Diagnostic yield of IgM ELISA showed 86.9% sensitivity and 100% specificity; IgG ELISA showed 96.8% sensitivity and 100% specificity. Both IgG and IgM ELISAs showed 100% Positive predictive value, 98.9% and 95.6% Negative predictive value respectively.

**Conclusion:**

A seroprevalence of 8.6% of human brucellosis was documented from this rural region. This is the first report from Wadi Al Dawaser. Frequent surveillance among risk group, vaccination of livestock, creating awareness and health education among the public and school children are recommended.

## INTRODUCTION

Brucellosis a significant zoonotic disease worldwide is also known as “Malta or Mediterranean fever”. The disease is caused by *Brucella*, which is small, fastidious Gram-negative coccobacilli. There are several species of *Brucella* which differ in their host range and degree of virulence. Human Brucellosis is caused by four important pathogenic species *B. melitensis*, *B. abortus*, *B. canis* and *B. suis*. *B. pinnipediae* and *B. cetaceae* are the recently recognized species to cause human infections.^1^

The natural reservoir of brucellosis is domestic animals. The infections are mostly present in goats, sheep, camel, cows, buffalo, horses, and pigs leading to animal abortion due to which production of milk gets lowered, causing an economic burden over the countries. In the natural animal host, the infection is highly contagious and animal-to-animal transmission is usually by venereal or ingestion of infected milk or tissue. High numbers of bacteria present in the unpasteurized raw milk and its dairy products such as cream, soft cheese, yogurt and, ice cream are the primary source of brucellosis in humans.^2^

Human brucellosis may also be contracted through inhalation of infectious aerosols, abrasions in the skin or direct contact with the conjunctiva. Handling animals or animal carcasses such as meat, blood, urine, vaginal secretions, placenta, and fetus are the primary sources of higher risk of direct zoonoses. These routes of infection are more important amongst people like agricultural workers, shepherds, butchers, veterinarians, and lab technicians. Though human-to-human transmission is rare, in case of transmission the probable routes are blood transfusion and bone marrow transplantation from infected donors, sexual intercourse, neonatal infection; trans-placental or during delivery or probably through breast milk.^3^

In human, the incubation period is 1-6 weeks or few months with an acute or chronic febrile illness. The disease has a variety of non-specific hematological abnormalities. The signs and symptoms may be clinically difficult to distinguish from a number of other infections such as typhoid fever, tuberculosis, dengue fever, and acute rheumatic fever. According to the World Health Organization (WHO), brucellosis is one of the most common zoonotic infections present worldwide with more than half a million new cases reported annually.^4^ The incidence and prevalence rates of brucellosis vary widely among Nations and the most massive disease burden lies in the Mediterranean and Middle East countries. Brucellosis is endemic in KSA and classified as a notifiable disease by Saudi Ministry of Health (MoH, 2006). It is estimated that the yearly incidence of brucellosis in the Kingdom is 12.5/100,000 population.^5^

*Brucella* species can survive for long periods in dung, soil, water, dust, aborted fetuses, dairy products, and meat. Human brucellosis is found to have a significant presence in the rural or nomadic areas where people live in close contact with animals.^3^ Wadi Al Dawaser is a small town in the central region of Saudi Arabia. The General Hospital provides tertiary care to people living in and around this region. Majority of the people in this part of the Kingdom are related to Bedouins or their expatriate shepherds, own flocks of camel, cattle and do agriculture. The floating population also consists of immigrants, for job and education purposes who live in rural areas with regular exposure to animals, or they may consume local animal products.

For the control of any infection, an epidemiological investigation from every part of the country is essential. Unfortunately, no reports were documented from this region of the Kingdom which is an important rural area and where people are still living the traditional lifestyle. Hence, this study was aimed to measure the seroprevalence of human brucellosis to provide baseline information as well as give the first indications about the extent of the problem in this study area. Further, to compare the different serological tests used for the diagnosis. This study will be the first to report the incidence of human brucellosis from this part of the Kingdom.

## METHODS

The study population includes patient attending the Wadi Al Dawaser general hospital with Pyrexia of Unknown Origin (PUO) and/or clinical characteristics of brucellosis throughout the study period of three years (2015-2018). Thirty-five personnel who were blood donors at the hospital’s blood bank were enrolled as the controls.

A standard pretested questionnaire was given to each patient suspected to have brucellosis in both Arabic and in the English language. The information like age, sex, nationality, education, residence (rural/urban), habit of consumption of raw milk or other milk products, contact with domestic animals, pregnancy status were collected. Both verbal and written consents were obtained from all the participants and patients (from parents in case of pediatric patients) before being involved in the study. The study was reviewed and approved by the Ethical Committee.

Blood sample (5ml) was collected from each patient and control. Serum was separated by centrifugation at 3000 rpm for five minutes. Sera were stored at -20°C, until tested for the presence of *Brucella* antibodies. All the positive sera were stored for further references.

For each sample, Rose Bengal Plate Agglutination Test (RBPT), Serum Agglutination Test (SAT), IgG and IgM ELISA were performed. The antigens for these tests were procured commercially and the procedures were carried out as per the manufacturer’s guidelines. Samples were considered as seropositive based on the positivity in one or more of these tests results.

RBPT was performed with commercial *Brucella* antigen (Crescent Diagnostics, KSA). On a clean glass slide, 30µl of test serum was mixed with 30µl of rose bengal antigen and mixed using a disposable stick. The slide was rotated manually for 5-6 minutes. The appearance of agglutinating clumps indicates positive reaction and the absence of clumps denotes a negative test. Known positive and negative serum was used as controls.^6^

SAT was performed in 2 ml small tubes. A series of nine tubes were labeled up in a rack, using a micropipette 1.9ml of 0.85% saline was dispensed in the first tube and 1ml of 0.85% saline into the remaining series of eight tubes. A volume of 0.1ml patient serum was added to the first tube, mixed, and then 1ml was transferred to the next tube. Further volumes of 1ml were transferred to subsequent tubes to give a series of doubling dilutions (from 1:20 to 1:1280) up to the eighth tube. The ninth tube was used as the saline control. An equal volume of standard *Brucella abortus* antigen was added to each tube and incubated at 37 °C in a water bath for 24 hours. After incubation, they were examined for agglutination. The last tube showing agglutination was taken as the titer value. A titer of ≥1:160 was considered as seropositive which would represent the presence of specific agglutination *Brucella* antibodies.^7^

The ELISA testing for IgG and IgM antibodies against *Brucella* species was performed using commercial reagents. The *Brucella* ELISA kit was procured from IBL, Hamburg, Germany. Separate microtiter plates for IgG and IgM were used. Briefly, 1µl of diluted serum was incubated at 37°C in the well for 1 hour and after buffer washing thrice, 100 µl of *Brucella* anti-IgG or anti-IgM conjugate was added and incubated for 30 minutes at room temperature. After, buffer washing, 100 µl of Tetra Methyl Benzidine (TMB) substrate was added and incubated for 15 minutes at room temperature. After incubation, 100 µl of stop solution was added, subsequently any blue colour developed during incubation turned into yellow. The optical density was measured with an ELISA reader at 450nm. The cut-off values were calculated as 10 U/mL as per manufactures guidelines. Interpretation of results was in units (U) using the formula, U= Patient (mean) absorbance value x 10 /Cut-off. Samples are positive if > 11 U, negative < 9 U and borderline 9-11 U. Re-testing was done for borderline samples to confirm positive or negative results.

The SPSS software version 20.0 was used to analyze the data collected. The tests were extracted using the Chi-square. The significant value of the P was determined to be < 0.001. The sensitivity, specificity, and negative and positive predictive values were calculated.

## RESULTS

For a period of three years consecutively from August 2015- August 2018, a total of 6721 blood samples from patients having presumptive diagnoses of brucellosis were tested. The number of samples collected during the year 2015-16 was 2086 of which 161 (7.7%) were positive. During 2016-17, 2416 samples were tested of which, 236 (9.8%) were positive and from 2017-18, a total of 2219 samples were tested of which 179 (8.1%) were positive for brucellosis. Thus of the total 6721 samples tested, 576 (8.6%) were positive for brucellosis (Table-I).

**Table-I:** Prevalence of human brucellosis in Wadi Al Dawaser region (2015-2018).

Year	Total no. of presumptive patients	Brucellosis Positive		Brucellosis Negative	

	(n)	(n)	(%)	(n)	(%)
2015-2016	2086	161	7.7	1925	92.3
2016-2017	2416	236	9.8	2180	90.2
2017-2018	2219	179	8.1	2040	91.9

The brucellosis seropositive patients were between 4 to 85 years of age group, with a mean age of 21.19 years and a standard deviation of ±14.39 years. Of these patients, 445 (77.3%) were male and 131 (22.7%) were female, with the male to female ratio of 3.3:1. Of the brucellosis positive cases, 473 (82.1%) were of Saudi Nationals and 103 (17.9%) were Non-Saudis. Regarding the level of literacy, 289 (50.2%) of the patients were illiterate, 109 (18.9%) completed high school and above. Demographic details of the Brucellosis positive patients are shown in Table-II. All the patients (100%) were residing in and around this rural area. Nineteen (14.5%) of the female patients were pregnant.

**Table -II:** Demographic details of the brucellosis positive patients (n=576)

Characteristic	(n)	(%)
***Gender***		
Male	445	77.3
Female	131	22.7
***Age Categories***		
<19 years	19	3.3
20-39 years	320	55.5
40-59 years	126	21.9
60-69 years	77	13.4
>70 years	34	5.9
***Nationality***		
Saudis	473	82.1
Expatriate	103	17.9
***Level of Education***		
Illiterate	289	50.2
Primary school	178	30.9
High school and above	109	18.9
***Duration of work (years)***		
<10	70	12.2
10-20	476	82.6
>20	30	5.2
***Direct contact with animal***		
Yes	485	84.2
No	91	15.8
***Abattoir Exposure***		
Yes	79	13.7
No	497	86.3
***Consumption of Unpasteurized/ raw milk & milk products***		
Yes	464	80.6
No	112	19.4

Duration of work years was 10-20 years in 476 (82.6%) followed by <10 years in 70 cases (12.2%). Investigation of cases by age, duration, and gender showed that working-age adolescent was mainly infected. Among the positive cases, 485 (84.2%) reported having direct contact with animals. Breeding, milking, parturient livestock were the different animal contacts reported in both the gender. Seventy-nine (13.7%) had abattoir exposure. Of the infected cases, 464 (80.6%) had the habit of consumption of raw milk and milk products. These were recognized as one of the possible risk factors for the brucellosis transmission in this study. Fig.1 (a) shows the month wise reported cases of human brucellosis. No significant seasonal variation was noticed. However, increased prevalence of 236 cases were recorded during 2016-17.

**Fig.1(a):**
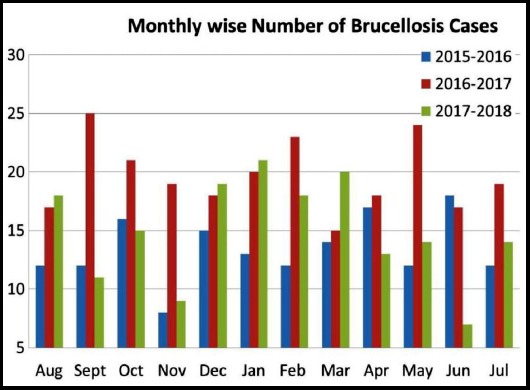
Month wise reported cases of human brucellosis (2015-2018)

In this study, of the 576 seropositive samples, RBPT identified 427 (74.1%), SAT titers identified 493 (85.6%) cases. IgM ELISA and IgG ELISA were positive for 501 (86.9%) and 558 (96.8%) cases respectively as shown in Fig.1 (b). All control sera samples were negative by the four tests.

**Fig.1(b):**
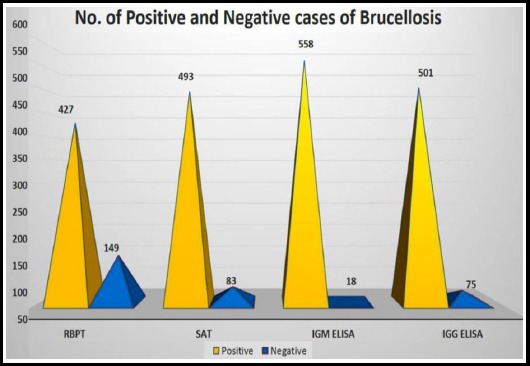
No. of positive and negative cases of brucellosis by different serological tests

One hundred and forty-nine samples negative by RBPT results were positive by SAT, IgM and/or IgG ELISA tests. Similarly, in comparing different SAT titers with ELISA positive cases, 159 samples with SAT titer of 1:160 and 96 samples with a titer of 1:320 were positive in both IgM and IgG ELISA. Of the 27 samples with SAT titer of 1:640, 21 were positive in IgM and all 27 in IgG ELISA. For the 15 samples with SAT titer of 1:1280, all were positive by IgG ELISA and only 2 positives by IgM ELISA (Table-III).

**Table-III:** Distribution of SAT titers and its comparison with ELISA results (n=493)

SAT titers	No. of Samples (n)	IgM ELISA		IgG ELISA	

		Positive(n)	Negative(n)	Positive(n)	Negative(n)
1:40	49	0	49	0	49
1:80	147	16	131	16	131
1:160	159	159	0	159	0
1:320	96	96	0	96	0
1:640	27	21	6	27	0
1:1280	15	2	13	15	0

Diagnostic yield of IgM ELISA showed 86.9% sensitivity and 100% specificity; IgG ELISA showed 96.8% sensitivity and 100% specificity. Both IgM and IgG ELISA showed 100% Positive predictive value and 95.6% and 98.9% Negative predictive value respectively.

## DISCUSSION

Brucellosis remains a major health problem in many parts of the world and is an important cause of acute febrile illness in the Middle East regions. However, according to WHO, brucellosis is listed as one of the seven neglected zoonotic diseases. Brucellosis is hyperendemic in Saudi Arabia with more than 8,000 cases reported each year to the public health authorities.^8^

In this study, the overall prevalence of human brucellosis in Wadi Al Dawaser region is 8.6%. In earlier reports from the Southwestern region, the seroprevalence rate was 16% and in the southern region was 19%.^8^,^9^ Similarly, in a study including 5507 individuals from the central region, the overall seropositive rate was reported as 48.5%.^10^ A prevalence rate of 2.6% was reported from North Western region.^11^ In another large-scale study analyzing the seroprevalence in different regions of Saudi Arabia, the highest prevalence of 20% and 18.3% were found in northern and southern regions respectively followed by 14% in the eastern and central region each, 11.6% in the western region.^12^ In a house to house survey of 4900 subjects in the southern region, 19.2% had exposure to *Brucella* antigen and 2.3% had active disease.^13^ These data show regional differences in the prevalence of antibodies to *Brucella* in countries in which the disease is endemic and the national seroprevalence of the disease for the Saudi population is 15%.^12^ Data from other neighboring countries shows a seroprevalence of 11.4% in Sudan^14^, 6.26 % in Egypt^15^ and 6.2% in Yemen.^16^

In this study, we observed brucellosis was more (77.3%) amongst male than (22.7%) female with a male to female ratio of 3.3:1. This is due to the fact that male is more involved in the risk of occupational exposure due to their direct contact with animals, meat, and milk products. Similar to our findings, other studies^8^,^17^ had shown a male predominance as well in the ratio of 2:1 and 3:1. In this study, 82.1% of the infected persons were Saudi nationals and the highest percentage of brucellosis recorded (55.5%) was in the age group of 20-39 years. This may be due to the fact that people in this working age group are more in contact with domestic animals like cattle breeding, farming, butchering and consume raw milk and dairy products. These were identified as one of the major risk factors (p<0.001) for brucellosis in this study. This result is in agreement with those reported elsewhere.^12^,^15^ However, it was found that children (<19years) were less frequently affected compared to adults, which is similar to our observation. Duration of work period 10-20 years has a significant role in getting the infection in 82.6% of the cases. No seasonal variation was noticed throughout the study. This is in agreement with other study findings from KSA.^8^,^18^ There is no seasonal influence on the incidence of brucellosis is noted in tropical and subtropical areas where animal breeding extends throughout the year.^4^ In addition, all the cases in our study were from rural area.

The diagnosis of brucellosis is subsequently more difficult on clinical symptoms alone and invariably requires lab testing particularly serological methods in endemic areas.^18^ In this study, for all the provisionally diagnosed cases serological tests such as RBPT, SAT, IgM and IgG ELISA were performed on each sample. IgM ELISA detected 96.8% of cases followed by IgG ELISA in 86.9%. Around 85.6% and 74.1% of the infected cases were detected by SAT and RBPT respectively. 19 Though the test is easy, simple and fast to perform, noticeably, for the 149 samples negative by RBPT, they were confirmed positive by ELISA. Thus the diagnosis could never be left out in these samples as RBPT indicated false-negative results and is low sensitive. This denotes a serious drawback of RBPT for *Brucella* diagnosis because an accurate diagnosis is crucial for prompt treatment. A number of positive cases would have been missed if RBPT test alone had been performed.

The total amount of agglutinating IgG and IgM antibodies are measured by SAT and is the most common acceptable serological diagnostic test for human brucellosis. In this study, the SAT has a higher specificity of 85.5%. However, interestingly for 16 samples with SAT titer of <1:80, they were positive by both IgM and IgG ELISA. These findings show that active brucellosis cannot be excluded in patients with SAT titers <1:160 especially in endemic areas where high clinical suspicion is seen. These data highlight the importance of using more than one serological test for diagnosis.

An overall concordant result between SAT titers and IgG and IgM ELISA titers was 88.3% in this study. Concordance results of 88.5 % and 91% were reported by others.^8^,^18^ In our study, discrepant results were obtained for 83 samples. Of the 83 samples negative by SAT titers, 18 were positive by both IgM and IgG ELISA and 57 samples positive by IgG ELISA. This is due to the fact that, in brucellosis patients, during the initial first week of infection IgM antibody levels may be detected which will reach its peak level after the fourth week. IgG antibodies which formed later will found mixed with IgM in the fourth week. Thus IgM exceeds IgG levels in the acute stage and IgG predominates over IgM in the chronic stage of infection. This could be the reason for our study findings.^18^ Further, it may be useful to screen acute sera for both IgM and IgG antibodies.

The diagnostic yield of ELISA tests showed that, IgG ELISA had 96.8% and IgM ELISA had 86.9% sensitivity. The specificity and positive predictive values of both were 100%. Both IgG and IgM ELISAs showed 100% Positive predictive value and 98.9% and 95.6% Negative predictive value respectively. This is in agreement with earlier studies that have identified ELISA as the best diagnostic test because of its high sensitivity.^8^,^18^,^19^ Detection of IgG antibodies is more sensitive than detection of IgM antibodies for diagnosing cases of brucellosis but specificity is comparable.^19^ Different studies have obtained different results regarding the sensitivity and specificity. Osaba et al reported sensitivities of *Brucella* ELISA IgG and IgM as 91% and 100%, respectively, while the specificity was 100% for both.^20^ Manthur et al reported ELISA sensitivity and specificity as 100% and 71.3% respectively.^18^ The commercial IgM ELISA showed low sensitivity of 60% in a study.^19^ Though ELISA is superior to other serological tests, the contradictions regarding the diagnostic ability of ELISA might be due to the usage of different commercial ELISA kits which varies depending on the manufacturer.^20^ Therefore, it is reasonable to further evaluate and standardize the test according to the various geographical regions and populations.^8^ In analyzing these findings in case of suspected brucellosis we recommend testing by both IgG and IgM ELISA for accurate diagnosis.

In the KSA, during the early 1980s, brucellosis emerged as a major public health problem. From then there has been a steady increase in the frequency over the past two decades. Recently, it was found that the incidence of reported cases of human brucellosis is slightly reduced from 2009 to 2012.^21^ This could be due to the concerted efforts of public health measures such as milk pasteurization, and livestock immunization.^22^ Since no effective vaccine is available for the prevention of brucellosis in human, reporting of brucellosis to health authorities from every part of the Kingdom is extremely important. This can be used to prioritize a disease control policy for brucellosis and to alert health staffs and the local community. During this study, health awareness about brucellosis and its risk factors was given to local public and to our university students as a part of community service.

## CONCLUSION

The current study shows a seroprevalence rate of 8.6% in Wadi Al Dawaser region. The disease is more prevalent among Saudi nationals and in the working age group. Contact with domestic animals and consumption of raw milk and milk products seems to be the major mode of transmission. In case of clinical suspicion, both IgM and IgG ELISA has diagnostic significance. Since person to person transmission is very rare, control of animal infection by vaccination, occupational and personal hygiene, farm sanitation and preventive measures can reduce disease incidence. Safe and effective vaccines for the prevention of human brucellosis are not generally available. However, continued efforts including creating awareness and health education among the public including young children in schools, frequent surveillance among the risk groups are warranted to eliminate the disease.

## References

[1] Bikas C, Jelastopulu E, Leotsinidis M, Kondakis X (2003). Epidemiology of human brucellosis in a rural area of north-western Peloponnese in Greece. Eur J Epidemiol.

[2] Smits HL, Kadri SM (2005). Brucellosis in India:a deceptive infectious disease. Indian J Med Res.

[3] Khan MY, Mah MW, Memish ZA (2001). Brucellosis in pregnant women. Clin Infect Dis.

[4] Corbel M (2006). Brucellosis in Humans and Animals:FAO, OIE, WHO.

[5] Ali IA, Zafer SA, Ahmed IA, Charlie PC (2015). Human brucellosis incidence trends in central Saudi Arabia (DawadmiGovernate). Int J Advanc Res.

[6] (1975). Laboratory techniques in brucellosis. World Health Organization &. Food and Agriculture Organization of the United Nations.

[7] Ariza J, Pellicer T, Pallares R, Foz A, Gudiol F (1992). Specific antibody profile in human brucellosis. Clin Infect Dis.

[8] Asaad AM, Alqahtani JM (2012). Serological and molecular diagnosis of human brucellosis in Najran, Southwestern Saudi Arabia. J Infect Public Health.

[9] Alballa SR (1995). Epidemiology of human brucellosis in southern Saudi Arabia. J Trop Med Hyg.

[10] Mofleh IAA, Aska AIA, Sekait MAA, Balla SRA, Nasser ANA (1996). Brucellosis in Saudi Arabia:Epidemiology in the Central Region.

[11] Al-Sekait MA (2000). Epidemiology of brucellosis in Al medina region, Saudi Arabia. J Family Community Med.

[12] Memish Z (2001). Brucellosis control in Saudi Arabia:prospects and challenges. J Chemother.

[13] Al-Sekait MA (1999). Seroepidemiology survey of brucellosis antibodies in Saudi Arabia. Ann Saudi Med.

[14] Tamador EA, Adil AR, Nageeb SS (2014). Seroprevalence of Human Brucellosis in Kuku DairyScheme, Khartoum State, Sudan. J Life Sci.

[15] Nawal HH, Wahid A (2012). Sero-prevalence of brucellosis in Egypt with emphasis on potential risk factors. World J Med Sci.

[16] Al-Haddad AM, Al-Madhagi AK, Talab AA, Al-Shamahy HA (2013). The prevalence of human brucellosis in three selected areas in Al-Dala'a governorate, Yemen. Faculty Sci Bull.

[17] Al-Tawfiq JA, Abukhamsin A (2009). A 24-year study of the epidemiology of human brucellosis in a health-care system in Eastern Saudi Arabia. J Infect Public Health.

[18] Mantur B, Parande A, Amarnath S, Patil G, Walvekar R, Desai A (2010). ELISA versus conventional methods of diagnosing endemic brucellosis. Am J Trop Med Hyg.

[19] Gomez MC, Nieto JA, Rosa C, Geijo P, Escribano MA, Munoz A, Lopez C (2008). Evaluation of seven tests for diagnosis of human brucellosis in an area where the disease is endemic. Clin Vacc Immunol.

[20] Araj GF, Kattar MM, Fattouh LG, Bajakian KO, Kobeissi SA (2005). Evaluation of the PANBIO Brucella immunoglobulin G (IgG) and IgM enzyme-linked immunosorbent assays for diagnosis of human brucellosis. Clin Diagn Lab Immunol.

[21] Bukharie HA (2009). Clinical features, complications and treatment outcome of Brucella infection:Ten years'experience in an endemic area. Trop J Pharm Res.

[22] Aloufi AD, Memish ZA, Assiri AM, McNabb SJ (2016). Trends of reported human cases of brucellosis, Kingdom of Saudi Arabia, 2004-2012. J Epidemiol Glob Health.

